# Inflammatory Biomarkers and Oral Health Disorders as Predictors of Head and Neck Cancer: A Retrospective Longitudinal Study

**DOI:** 10.3390/ijms26052279

**Published:** 2025-03-04

**Authors:** Amr Sayed Ghanem, Kitti Sipos, Ágnes Tóth, Attila Csaba Nagy

**Affiliations:** 1 Department of Health Informatics, Faculty of Health Sciences, University of Debrecen, 4028 Debrecen, Hungary; aghanem@etk.unideb.hu; 2 Department of Operative Dentistry and Endodontics, Faculty of Dentistry, University of Debrecen, 4032 Debrecen, Hungary; sipos.kitti@dental.unideb.hu; 3 Department of Integrative Health Sciences, Faculty of Health Sciences, University of Debrecen, 4028 Debrecen, Hungary; toth.agnes@etk.unideb.hu

**Keywords:** C-reactive protein, CRP, eGFR, estimated glomerular filtration rate, head and neck cancers, oral cavity cancer, oral squamous cell carcinoma, malignant

## Abstract

Head and neck cancers (HNCs) are often diagnosed late, leading to poor prognosis. Chronic inflammation, particularly periodontitis, has been linked to carcinogenesis, but systemic inflammatory markers remain underexplored. This study was the first to examine whether elevated C-reactive protein (CRP) can serve as a cost-effective adjunct in HNC risk assessment, alongside oral health indicators. A retrospective cohort study analysed 23,742 hospital records (4833 patients, 2015–2022) from the University Hospital of Debrecen. HNC cases were identified using ICD-10 codes, with CRP and periodontitis as key predictors. Kaplan–Meier survival analysis, log-rank tests, and Weibull regression were used to assess risk, with model performance evaluated via AIC/BIC and ROC curves. Periodontitis was significantly associated with HNC (HR 5.99 [1.96–18.30]), while elevated CRP (>15 mg/L) independently increased risk (HR 4.16 [1.45–12.00]). Females had a significantly lower risk than males (HR 0.06 [0.01–0.50]). CRP may serve as a cost-effective, easily accessible biomarker for early HNC detection when combined with oral health screening. Integrating systemic inflammation markers into HNC risk assessment models could potentially improve early diagnosis in high-risk populations.

## 1. Introduction

Head and neck cancer (HNC), which includes malignancies of the lip and oral cavity, is among the most prevalent cancers worldwide, with lip and oral cavity cancers ranking 13th [1] globally. It is more common in men and in the elderly population [1]. Approximately 90% of HNCs are squamous cell carcinomas, while the remaining 10% include sarcomas, melanomas, lymphomas, salivary gland tumours, and odontogenic tumours [2]. Sores or lumps on the lips or inside the mouth are common and simple indicators of neoplasms [3]. Despite the fact that the disease is easily recognisable, many people seek medical help only at advanced stages, which plays a crucial role in the high mortality rate of the disease, which is nearly 50% [3]. In order to enhance prognosis and reduce the number of fatal cases, early diagnosis is essential [4]. The most common methods for diagnosis are visual inspection and palpation, which may be accompanied by biopsy and histological analysis (e.g., toluidine staining). Additionally, imaging techniques may also be involved in the detection and precise staging of the primary tumour(s) or regional lymph nodes [5]. In cases of identified disorders, the conventional therapy is surgical removal, potentially supported by radiation and/or chemotherapy or other postoperative adjuvants [6,7].

There is a large amount of evidence that periodontitis is associated with an increased risk of developing certain non-communicable chronic diseases [8,9,10], such as several types of cancers, including HNCs [11]. Periodontitis is the inflammation of the periodontium [12], which is typically a chronic condition and followed by clinical attachment and alveolar bone loss. It affects tooth location, leading to drifting, and tooth instability, resulting in increased mobility [13]. Periodontitis may include periodontal pocketing, recession, or enlargement of the gingiva and bleeding, and it is a major cause of tooth loss in society [12,13]. It is estimated that approximately 19% of the adult population or over 1 billion people is affected by severe periodontal disease [1]. Due to the multifactorial nature of the disease [9] a number of factors have been identified as associated with the higher likelihood of periodontal disease, which involves the dysbiosis of the oral microbiota as a major causative factor [11].

The composition of the oral microbiome can be altered by different lifestyle habits such as cigarette smoking, alcohol consumption, diet, and poor oral health, and it can be influenced by several medical conditions as well, e.g., diabetes mellitus or chronic kidney disease [14,15,16,17], resulting in the overpopulation of certain pathogenic microorganisms [18] such as the Gram-negative and anaerobic bacterial species, Porphyromonas gingivalis, Aggregatibacter actinomycetecomitans, and Fusobacterium nucleatum [9], among which Porphyromonas gingivalis is considered to be the primary causative bacterial strain [19]. Persistent infections have the ability to induce tumorigenesis; however, it is important to note that bacteria work synergistically, and one bacterium is insufficient to determine the entire process in an organism [20]. The transcription factor nuclear factor kappa B (NF-κB) is a central regulator of inflammation-driven carcinogenesis [21]. Pathogenic bacteria release endotoxins such as lipopolysaccharide (LPS), which activates toll-like receptor 4 (TLR4), initiating NF-κB-mediated signalling cascades that promote cytokine release [14]. This pathway drives cellular transformation, proliferation, apoptosis evasion, invasion, angiogenesis, and metastasis—hallmarks of cancer progression [21]. A positive feedback loop between NF-κB and pro-inflammatory mediators, particularly tumour necrosis factor-alpha (TNF-α), interleukin-6 (IL-6), and IL-1β, further amplifies chronic inflammation, perpetuating a tumour-promoting microenvironment [22].

Inflammatory factors can result in periodontal tissue damage [9,11,23] and induce the production of C-reactive protein (CRP) by hepatocytes after systemic dispersion [24,25]. IL-6 is the major cytokine inducing the expression of CRP during the acute phase response [26] by transcription factor signal transducer and activator of transcription 3 (STAT3); additionally, IL-1β acts as a synergistic enhancer in the process [27,28]. CRP secreted from hepatocytes is a pentameric molecule and circulates in the systemic vasculature [29]. After binding to lipid rafts of cells engaged in inflammatory reactions, it dissociates to a highly active monomeric form and interacts with many cell types at the sites of inflammation and the components of the extracellular matrix (ECM) [29]. Pentameric CRP is a measurable quantity, suitable for diagnostic testing [29]. Epidemiological studies have reported that serum CRP levels are elevated in patients suffering from chronic periodontitis. CRP is currently regarded as one of the most relevant biomarkers of systemic inflammation [25].

Emerging evidence suggests that ferroptosis, an iron-dependent form of regulated cell death, plays a pivotal role in oral squamous cell carcinoma (OSCC) pathogenesis and progression. The dysregulation of ferroptosis pathways contributes to tumour survival, immune evasion, and therapy resistance, underscoring its potential as both a biomarker and therapeutic target [30]. Notably, pro-inflammatory cytokines, particularly interleukin-6 (IL-6), promote ferroptosis resistance via JAK2/STAT3-mediated upregulation of xCT (SLC7A11), an antiporter critical for glutathione homeostasis, reinforcing the role of chronic inflammation in OSCC carcinogenesis [31].

Ferroptosis-related genes (FRGs) and their associated non-coding RNAs have been identified as prognostic biomarkers in OSCC, with glutathione peroxidase 4 (GPX4) and adipocyte enhancer-binding protein 1 (AEBP1) serving as key mediators of ferroptosis suppression [32]. In addition, FTH1 (ferritin heavy chain 1), a critical iron storage protein, has been implicated in OSCC proliferation and epithelial–mesenchymal transition (EMT), with its inhibition inducing ferroptotic cell death, highlighting its therapeutic potential [33].

Given that HNC can remain asymptomatic for long periods, its early detection remains a major clinical challenge [3], contributing to delayed diagnosis and poor prognosis [4]. While chronic inflammation, particularly periodontitis [11], has been implicated in oral carcinogenesis, limited research has evaluated systemic inflammatory markers as potential adjuncts in HNC risk assessment. Despite evidence linking periodontitis to malignant transformation, no widely accessible biomarker has been integrated into routine screening for HNC risk stratification. This study aimed to bridge this gap by investigating whether CRP, a readily available inflammatory biomarker, could serve as a cost-effective tool for early detection, alongside oral health indicators.

Since longitudinal investigations are essential to establish causalities, our study aimed to assess the associations of CRP and periodontitis with HNC while identifying other potential predictors of the disease, including the estimated glomerular filtration rate (eGFR), since patients with chronic kidney disease might be more susceptible to oral health issues due to oral microbial changes. Furthermore, apart from periodontitis, additional oral conditions were investigated, such as gingival disorders, dental development disorders, eruption issues, hard tissue, pulp, periapical tissue diseases, dental caries that can act as plaque retention factors and promote the growth of anaerobic bacteria, and gingivitis, which is a mild form of periodontal disease and, if untreated, might also lead to periodontitis and, ultimately, malignancy [30].

## 2. Results

### 2.1. Baseline Characteristics

The baseline characteristics of the 4833 participants are summarised in Table 1. The mean age was 50.43 years (SD = 15.40), with a median of 51 years (IQR = 40–62). The cohort consisted of 2193 males (45.40%) and 2637 females (54.60%). Periodontitis was present in 1514 participants (31.33%). Among those with available data, elevated CRP levels (>15 mg/L) were observed in 102 participants (25.69%), while 295 (74.31%) had normal levels (≤15 mg/L). Kidney disease (eGFR < 60 mL/min/1.73 m^2^) was reported in 78 participants (19.75%) out of those with available eGFR data. Disorders of tooth development and eruption (K00) were present in 66 participants (1.37%), Embedded and impacted teeth (K01) were noted in 40 participants (0.83%). Dental caries (K02) affected 135 participants (2.79%), other diseases of hard tissues of teeth (K03) was reported in 770 participants (15.93%), and diseases of pulp and periapical tissues (K04) were documented in 147 participants (3.04%). Other disorders of gingiva and edentulous alveolar ridge (K06) were observed in 77 participants (1.59%), and other specified disorders of teeth and supporting structures (K08) were present in 167 participants (3.46%).

### 2.2. Survival Distributions and Log-Rank Test Results

The results of the log-rank test, comparing survival distributions across the different categories, are summarised in Table 2. A significant difference in survival probabilities was observed for periodontitis (*p* < 0.001), with 114 events in participants with periodontitis compared to an expected 38.15. Elevated C-reactive protein levels (>15 mg/L) were also significantly associated with survival outcomes (*p* = 0.007), with 20 observed events versus 8.1 expected. Gender showed a strong association (*p* < 0.001), with males experiencing 130 events compared to an expected 73.5, while females had 43 events compared to the 99.5 expected. Among dental conditions, significant associations were identified for disorders of tooth development and eruption (K00, *p* = 0.042), embedded and impacted teeth (K01, *p* = 0.011), dental caries (K02, *p* < 0.001), other diseases of hard tissues of teeth (K03, *p* < 0.001), and other specified disorders of teeth and supporting structures (K08, *p* < 0.001). Conversely, no significant differences were observed for diseases of pulp and periapical tissues (K04, *p* = 0.74), other disorders of gingiva and edentulous alveolar ridge (K06, *p* = 0.971), or eGFR (*p* = 0.489).

### 2.3. Cumulative Hazard Analysis

**Figure 1:**
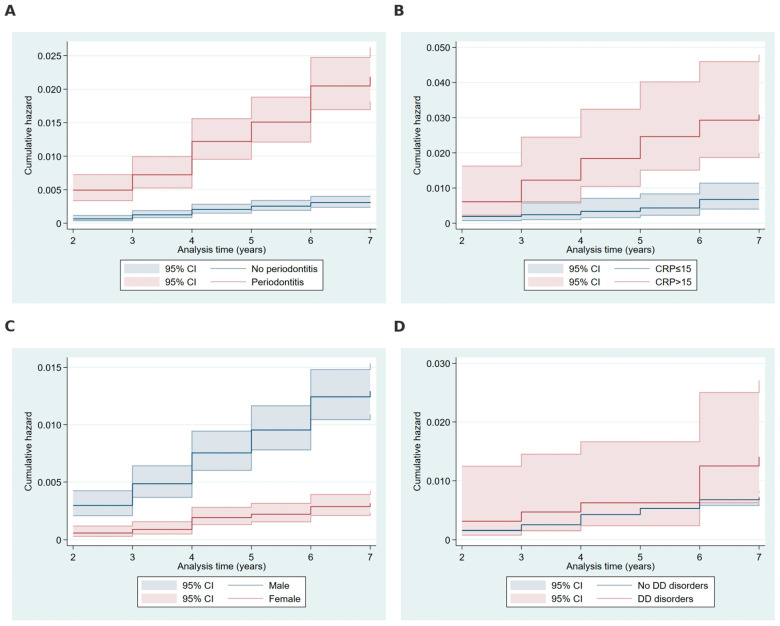
Cumulative hazard plots by periodontitis, CRP levels, gender, and dental developmental disorders. Note: Cumulative hazard functions illustrating the relationships between time to head and neck cancer diagnosis and key variables: (**A**) periodontitis (yes vs. no), (**B**) C-reactive protein levels (>15 mg/L vs. ≤15 mg/L), (**C**) gender (male vs. female), and (**D**) dental developmental (DD) disorders (present vs. absent). Hazard functions are stratified by categories with 95% confidence intervals (shaded areas), calculated using the Nelson–Aalen estimator. CRP, C-reactive protein; DD disorders, dental developmental disorders.

The cumulative hazard curves (Figure 1) illustrate the temporal progression of oral cancer risk across key variables identified as significant predictors in the log-rank tests. Figure 1A highlights the finding that participants with periodontitis exhibited higher cumulative hazards for HNC compared to those without periodontitis. Similarly, participants with elevated C-reactive protein levels (>15 mg/L) demonstrated higher cumulative hazards compared to those with normal CRP levels (Figure 1B). Gender-stratified cumulative hazard curves (Figure 1C) indicate that male participants had consistently higher cumulative hazards for HNC than females over the follow-up period. Lastly, Figure 1D depicts the cumulative hazards associated with dental developmental (DD) disorders, showing that participants with DD disorders had a markedly increased cumulative hazard compared to those without such disorders.

**Figure 2:**
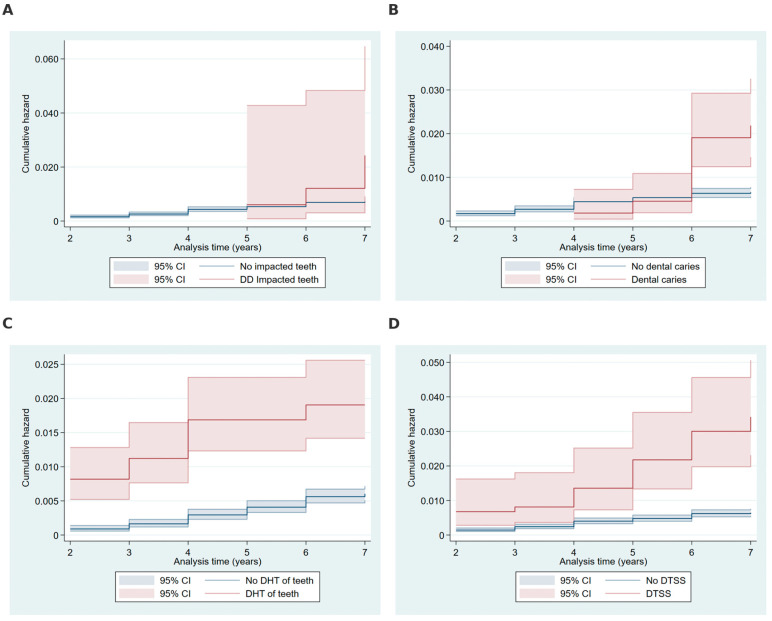
Cumulative hazard plots by embedded and impacted teeth, dental caries, disease of hard tissue of teeth, and disorders of teeth and supporting structures. Note: Cumulative hazard functions depicting the association between time to head and neck cancer diagnosis and key dental conditions: (**A**) embedded and impacted teeth (present vs. absent), (**B**) dental caries (present vs. absent), (**C**) disease of hard tissue (DHT) of teeth (present vs. absent), and (**D**) disorders of teeth and supporting structures (DTSS) (present vs. absent). Hazard functions are stratified by condition categories with 95% confidence intervals (shaded areas), calculated using the Nelson–Aalen estimator. DHT, disease of hard tissue; DTSS, disorders of teeth and supporting structures.

In Figure 2A, participants with embedded and impacted teeth exhibited a noticeably higher cumulative hazard over the follow-up period compared to those without this condition. Figure 2B demonstrates that participants with dental caries experienced an elevated cumulative hazard, particularly in later years of follow-up, compared to those without caries. Figure 2C illustrates the effect of disease of hard tissue (DHT) of teeth, where individuals with this condition showed a marked increase in cumulative hazard over time relative to those without DHT. Finally, Figure 2D highlights a significant cumulative hazard increase among participants with disorders of teeth and supporting structures (DTSSs) compared to those without these disorders, with a sharper rise observed during the later years of follow-up.

### 2.4. Weibull Regression Results

Participants with periodontitis demonstrated a markedly increased hazard, with an HR of 5.99 (95% CI: 1.96–18.30, *p* = 0.002). Similarly, elevated CRP levels (>15 mg/L) were associated with an increased hazard of HNC, with an HR of 4.16 (95% CI: 1.45–12.00, *p* = 0.008). Gender was also a significant predictor; females had a reduced hazard compared to males, with an HR of 0.06 (95% CI: 0.01–0.50, *p* = 0.009). Participants with embedded and impacted teeth (K01) had a markedly elevated hazard of HNC (HR = 12.52, 95% CI: 2.48–63.18, *p* = 0.002), the highest among all examined oral conditions. Although disorders of tooth development and eruption (K00) approached significance (HR: 3.97, 95% CI: 0.88–17.92, *p* = 0.073), it did not meet the threshold for statistical significance. Other oral health conditions, including dental caries (K02), other diseases of hard tissues of teeth (K03), diseases of pulp and periapical tissues (K04), and other specified disorders of teeth and supporting structures (K08), were not significantly associated with HNC hazard (all *p* > 0.05). Age, as a continuous variable, showed no significant association with HNC hazard (HR: 0.98, 95% CI: 0.95–1.02, *p* = 0.347). Kidney disease, defined by eGFR < 60 mL/min/1.73 m^2^, was not significantly associated with the hazard of HNC (HR: 0.95, 95% CI: 0.35–2.61, *p* = 0.919) (Table 3).

In the Weibull regression analysis, the shape parameter (*p*) outlined in Table 4 was estimated to be 2.20 (95% CI: 1.39–3.49). This value indicates that the hazard of developing HNC increases over time, as *p* > 1 reflects a positive ageing effect. The increasing hazard aligns with the biological plausibility of cumulative risk exposure and age-related changes contributing to disease progression.

### 2.5. Model Validation and Discrimination Metrics

**Figure 3:**
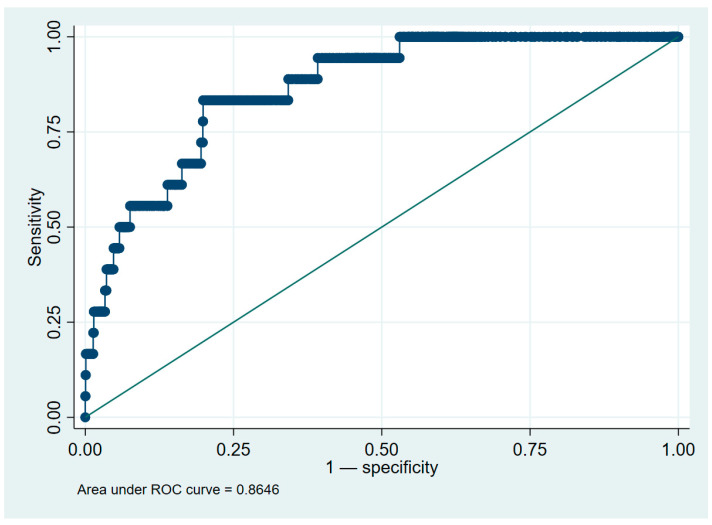
Receiver operating characteristic (ROC) curve of the Weibull regression model for predicting head and neck cancer. Note: The ROC curve shows an AUC of 0.8646, demonstrating excellent model discrimination.

The discriminative performance of the Weibull regression model was evaluated using the area under the ROC curve and Harrell’s concordance statistic. The ROC curve yielded an area under the curve (AUC) of 0.8646 (95% CI: 0.7933–0.9359), indicating excellent predictive accuracy (Figure 3). Harrell’s c-statistic confirmed the model’s robustness with a concordance coefficient of 0.8646 (95% CI: 0.7953–0.9339). These metrics demonstrate the model’s strong ability to distinguish between participants who developed HNC and those who did not during the follow-up period.

**Figure 4:**
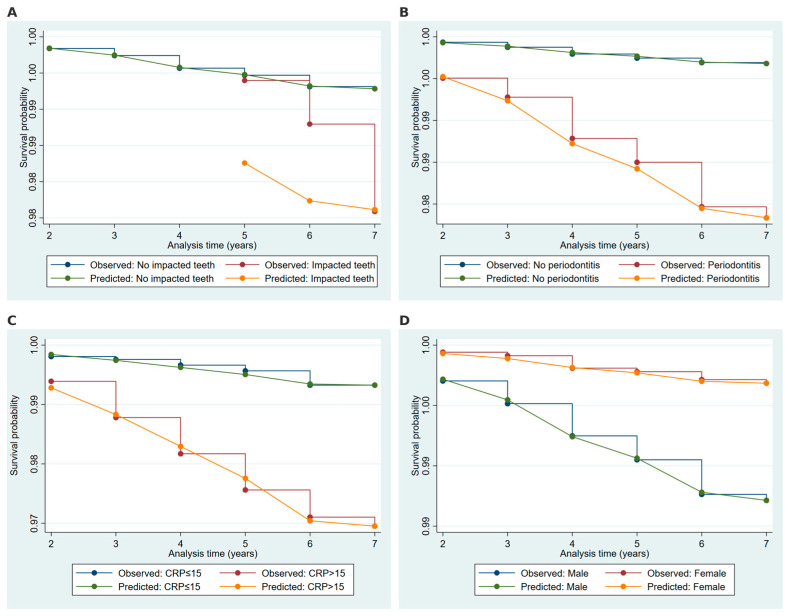
Kaplan–Meier survival curves stratified by significant predictors. Kaplan–Meier survival curves depicting observed and Weibull model-predicted probabilities over time. Stratification is based on significant covariates: (**A**) impacted teeth, (**B**) periodontitis, (**C**) C-reactive protein (CRP) levels, and (**D**) gender.

Kaplan–Meier curves were constructed to illustrate the observed and predicted survival probabilities over time, stratified by significant covariates identified in the Weibull regression model. These curves were stratified by the presence or absence of embedded or impacted teeth (Figure 4A), periodontitis (Figure 4B), CRP levels (Figure 4C), and gender (Figure 4D). Panel A compares participants with and without embedded or impacted teeth, showing a steeper decline in survival probabilities among those with missing teeth, with Weibull model predictions closely aligning with observed data. Panel B examines participants with and without periodontitis, revealing a marked reduction in survival probability over time for those with periodontitis compared to their counterparts. Panel C presents survival probabilities based on CRP levels, where participants with high CRP levels (>15 mg/L) experienced noticeably lower survival probabilities, while those with normal CRP levels maintained higher probabilities throughout the follow-up period. Lastly, Panel D stratifies survival by sex, demonstrating that male participants exhibited lower survival probabilities than females, with the Weibull model predictions accurately reflecting observed trends in all cases.

## 3. Discussion

The objective of the present study was to investigate the association between predictors of HNC, with a focus on identifying any predictive factors, such as CRP or eGFR, which have not been previously investigated in the context of such type of malignancy as a potential method for secondary prevention. This study is based on a large clinical database of cases and makes a significant contribution to the identification of risk factors by examining several concurrent factors. The study is of particular significance in the context of cancer research, as it focuses on a persistent and significant public health concern in Hungary. Specifically, HNC is the eighth most prevalent cancer type among both sexes in the country [34]. Whilst encouraging signs are evident in terms of a slight decline in the incidence and mortality rates of the aforementioned cancer types in the country, Hungary remains among the European countries with the highest rates of HNC including oral cavity cancers [34,35].

Risk factors for HNC represents a significant field of research [36,37]. In this context, Hungary exhibits comparatively weaker performance with regard to smoking and alcohol consumption in comparison to other European Union countries. Conversely, the country displays comparatively stronger rates of vaccination against the human papilloma virus (HPV) [38].

A review of the literature reveals a clear pattern indicating that HNC is more prevalent in males than in females [39,40], a trend that is also supported by Hungarian data [34]. The present study also demonstrated that the risk of developing HNC was lower for females than for males. This phenomenon may be attributed to disparities in tumour biology and hormonal influences, which render men more susceptible to the development of such malignancies. It has also been observed that women in Hungary exhibit superior oral hygiene [41] and men are more likely to engage in high-risk behaviours, such as smoking and excessive alcohol consumption. However, there is an increasing concern that the incidence of lung cancer among women is rising as a consequence of changes in smoking habits [34], which may also contribute to an increase in the incidence of HNC among women in the future. A previous study conducted at the Department of Oral Surgery of the University of Debrecen revealed that the majority of patients diagnosed with oral squamous cell carcinoma (OSCC) were smokers (65.5%) and reported alcohol consumption (75.5%) at the time of diagnosis. Only 12.6% of cases were found to have acceptable dental status [42].

The prevalence of dental caries in Hungary is indeed high [41], yet the present study found no association with oral cavity cancer. Tezal et al. found that patients with a high caries incidence, i.e., those with a predominantly cariogenic oral flora, displayed a reduced propensity for HNC development. Cariogenic bacteria have been shown to induce a Th1-mediated immune response, which has been demonstrated to result in a tumour suppressor effect [43].

In addition to dental caries, periodontal disease is the most prevalent bacterial infection of the oral cavity [44]. The present study also revealed a very high prevalence (31%) of periodontitis among the study participants. As demonstrated by numerous human and murine studies, the idea that Th1 cells and their cytokines characterize early/stable periodontal lesions is corroborated. By contrast, Th2 cells are implicated in the progression of the disease [45]. Periodontopathogenic flora has been observed to elicit a Th2/Th17 immune response, which has been associated with an elevated risk of tumour development [43]. The presence of Th2 cells and the cytokines they secrete has been linked to a poor prognosis in various malignancies, including HNC [46,47]. It has been hypothesized that Th2 responses accelerate the growth of tumours by inhibiting Th1-mediated anti-tumour action and boosting angiogenesis. However, findings suggest a more complex function for Th2 cells. Despite the identification of several key pathways, the precise mechanisms by which Th2 cells promote tumour growth remain to be elucidated [47]. Th17 cells, which originate from CD4+ T cells, have been shown to play a pivotal role in the progression and regulation of periodontal disease, with the cytokines they secrete (mainly IL-17 and IL-22) being a key factor in this process [48,49]. Th17 cells have also been shown to play a significant role in the promotion of inflammation in a variety of pathophysiological situations, including HNC [50]. These cells exhibit remarkable plasticity, allowing them to exhibit different phenotypes in the cancer microenvironment. The role of Th17 cells in cancer is multifaceted and dependent on the unique characteristics of the tumour. These cells can promote tumour progression through immunosuppressive activities and angiogenesis but also mediate anti-tumour immune responses through the use of immune cells in the tumour environment or by directly converting to the Th1 phenotype and producing interferon-γ (IFN-γ) [47]. The findings of this study demonstrated a substantial correlation between the occurrence of HNC and periodontitis, aligning with the findings of other researchers [51,52,53,54,55]. Therefore, dentists should consider that patients with periodontitis, especially those with coexisting lifestyle risk factors [41], have been linked to an increased risk of HNC.

The examination of factors associated with dental status revealed in this study that embedded and impacted teeth (K01) exhibited a notable oral health variable associated with an increased hazard. This represents a previously unreported association in the literature. This phenomenon can be attributed to the constant irritation and infection that occurs around the impacted teeth, which can result in chronic inflammation and persistent infections. A significant body of evidence exists, which indicates a clear association between chronic inflammation and cancer [56]. There is also a possibility that cystic lesions develop around the region of impacted wisdom teeth, which may subsequently transform into tumours [57]. This transformation carries with it a risk of malignancy [58]. A definitive causal relationship between impacted wisdom teeth and HNC has yet to be established. Consequently, these conditions can serve to increase the overall risk factors associated with cancer. Nevertheless, cysts and tumours have been observed in a small percentage of patients with impacted wisdom teeth. As a consequence, prophylactic removal of the wisdom tooth is not considered necessary [57]; however, subsequent follow-up is recommended.

The gold standard for the diagnosis of HNC is tissue biopsy and subsequent histological evaluation [4]. Nevertheless, there is the possibility of false-positive or false-negative biopsy results. HNC is often diagnosed at an advanced stage, which has a negative impact on patient survival rates [42]. There is mounting evidence to support the hypothesis that chronic inflammation is a contributing factor to the development of cancer [53]. Consequently, a significant number of studies have been conducted with the objective of identifying easily accessible early diagnostic biomarkers [4,59]. For instance, CRP has been shown to have elevated levels that correlate positively with tumour status [59,60]. The precise mechanisms underlying the association CRP levels and the survival of patients diagnosed with HNC remain to be elucidated; however, the following factors may be contributing factors [60]. It has been hypothesized that chronic inflammation may play a role in the development and progression of HNC [4]. Inflammation exerts a significant influence on the composition of the tumour microenvironment. In response to inflammation, CRP is synthesized in the liver by the stimulation of IL-6. The IL-6 has been demonstrated to accelerate angiogenesis and inhibit ferroptosis, thus promoting the progression and metastasis of tumours [31,60]. Cancer cells can produce a number of chemokines and cytokines, which in turn results in an increase in the serum CRP level [60]. Moreover, a higher prevalence of tobacco use, alcohol consumption, and poor oral hygiene has been observed in patients with HNC, which has also been demonstrated to elevate CRP levels [61,62,63]. The role of the CRP is of significance, given that it has been identified as a potential mediator of carcinogenesis and cancer progression via the activation of the FcgRs/MAPK/ERK, FcgRs/NF-kB/NLRP3, and FcgRs/IL-6/AKT/STAT3 pathways [60].

This is the first study of its kind to attempt to find a correlation in HNC patients prior to cancer diagnosis using pre-diagnosis CRP data. The Weibull regression results indicate that elevated CRP levels (>15 mg/L) are associated with an increased hazard of HNC. Serum CRP level can be measured in a simple and repeatable manner, and the associated financial cost is reasonable. Therefore, it could be regarded as a routine clinical marker in patients with HNC [59,60]. Salivary CRP has recently attracted considerable attention from the scientific community. A large body of research has demonstrated the correlation between CRP levels in blood and saliva, suggesting that salivary CRP can serve as a reliable surrogate marker for serum CRP as an indicator of oral inflammation. The advent of novel technologies capable of detecting CRP in saliva underscores its potential as a diagnostic instrument for various oral inflammatory and immune conditions, including periodontitis and HNC, given the non-invasive nature of saliva collection [63].

In addition to CRP, renal function, measured via eGFR, was investigated for its potential role in modulating cancer risk, given its known association with systemic inflammation. To date, no article has specifically examined the association between reduced eGFR and HNC. The present article therefore constitutes pioneering work in this regard. As demonstrated by other researchers, the incidence rate of cancer was found to be comparatively higher in patients suffering from chronic kidney disease than in the general population [64,65]. In contrast, Wong et al. did not observe an association between reduced renal function and the overall risk of cancer [66]. Whilst no significant correlation was identified in the present study with eGFR, this may be attributable to the complex relationship between renal function and systemic inflammation.

In accordance with the projected changes in population growth and ageing, and assuming that overall cancer rates remain constant, Bray et al. predict that over 35 million new cancer cases will occur in the year 2050. This figure signifies a 77% increase on the 20 million cases estimated in 2022 [40]. The authors therefore consider research into the identification of risk factors and early detection to be of the utmost importance. Such research may contribute to a reduction in the alarming figure in the future.

### 3.1. Future Directions

The present study identified the presence of embedded and impacted teeth (K01) as a significant oral health variable associated with an elevated HNC risk. This finding signifies a previously unreported association in the literature and could serve as a basis for further research.

Elevated levels of CRP (>15 mg/L) have also been demonstrated to be associated with an increased hazard of HNC. However, further investigation is required to ascertain whether elevated CRP levels occur prior to the biological onset of cancer or whether they act as a risk factor for its onset.

The absence of a substantial correlation between eGFR and the variables under investigation in the present study may be ascribed to the complex relationship between renal function and systemic inflammation. Further research is required to ascertain the underlying mechanisms.

### 3.2. Strengths and Limitations

A key strength of this study is its utilisation of real-world clinical data collected over a seven-year follow-up period, allowing for a comprehensive evaluation of the natural history and progression of HNC. This study benefits from a substantial sample size, which enhances statistical power and reliability. Additionally, the inclusion of biomarker data obtained through laboratory assessments provides an objective measure of systemic health, strengthening the validity of associations examined. Diagnoses were established by physicians based on ICD-10 codes, ensuring clinical applicability and diagnostic accuracy. The longitudinal study design facilitated the assessment of temporal and potential causal relationships, while the implementation of robust time-to-event analytical models enhanced methodological rigour by accounting for both systemic and intraoral health parameters.

However, several limitations should be acknowledged. The dataset originated from a single-centre clinical registry, which may restrict the external validity and generalizability of findings to broader or more diverse populations. Moreover, while ICD-10 coding ensures diagnostic precision, it lacks granularity regarding disease severity or staging, limiting a more detailed assessment of disease burden. The absence of key socioeconomic and demographic variables precluded an in-depth evaluation of their potential confounding effects. Smoking, which is a well-documented risk factor for HNC, was not accounted for due to the unavailability of smoking status within the clinical database, which might have influenced the robustness of the observed associations. This study is also a retrospective observational study; therefore, inherent biases related to data availability, selection, and recording could not entirely be excluded. Lastly, given the observational nature of this study, unmeasured confounding variables remain a potential source of bias, necessitating cautious interpretation of causal inferences.

## 4. Materials and Methods

This study involving human participants was approved by the Ethics Committee at the University of Debrecen (Approval Number: 6054-2022) on 20 April 2022. This study was conducted in accordance with relevant local laws and institutional guidelines. It utilised secondary analysis of pre-anonymized, de-identified hospital records, ensuring that no identifiable personal information was accessible during the research process.

### 4.1. Data Cleaning and Processing

This study utilised a retrospective longitudinal design based on hospital records collected between 2007 and 2022 by the University Hospital of Debrecen in Hungary. The initial dataset consisted of 37,164 hospital records, representing visits of participants between years 2007 and 2022 diagnosed with various conditions by physicians using ICD-10 codes. To improve data quality and focus on the study period with the most complete and precise records, all data prior to 2015 were excluded. This exclusion was justified by the minimal number of HNC cases recorded before 2015 and the widespread missing laboratory data. After this step, 23,742 records remained, covering visits from 2015 to 2022.

Participants with a diagnosis of HNC (ICD-10 codes: C00–C10, C14) at or prior to baseline were excluded to ensure all individuals were free of the failure event at the start of follow-up. Head and neck cancer cases were identified using the variable “HNC”, defined as present when any of the following ICD-10 codes were recorded: C00, C01, C02, C03, C04, C05, C06, C07, C08, C09, C10, or C14. For periodontitis, a binary variable “periodontitis” was created based on the presence of any of the following ICD-10 codes: K05, K05.2, K05.3, K05.4, K05.5, or K05.6.

Participants were eligible for inclusion if they had at least one documented dental or oral health-related diagnosis within the hospital’s electronic health records (EHRs) between 2015 and 2022, ensuring the availability of longitudinal data for oral–systemic health assessment. Only individuals with confirmed head and neck cancer (HNC; ICD-10: C00–C14) diagnosed after the baseline dental visit were included in survival analyses to mitigate immortal time bias. To ensure data completeness and feasibility of time-to-event modelling, participants were required to have a minimum of two years of follow-up within the hospital database. Only adult patients (≥18 years old) were eligible, as paediatric and adolescent populations follow distinct clinical trajectories in both oral pathology and malignancy risk.

Exclusion criteria encompassed individuals with a prevalent diagnosis of HNC at baseline, ensuring that all participants were free from the failure event at study entry. Participants with insufficient follow-up data (i.e., lost to follow-up before two years) were excluded to minimise bias from incomplete risk estimation. Given the study’s focus on periodontitis and its systemic implications, patients with only secondary or incidental dental diagnoses (e.g., traumatic dental injuries without evidence of chronic inflammation) were excluded to maintain clinical homogeneity. Additionally, participants with systemic inflammatory conditions unrelated to oral health (e.g., autoimmune diseases, systemic lupus erythematosus, rheumatoid arthritis) were excluded to prevent confounding effects on systemic inflammatory markers. Finally, patients with multiple healthcare system transfers or fragmented EHRs were excluded to minimise selection and information bias due to incomplete medical histories.

The follow-up time for each participant was calculated as the difference between their baseline year and either the year of diagnosis (for those who developed oral cancer) or the study’s endpoint in 2022 (for censored observations). The follow-up exit year was defined as the diagnosis year for participants with oral cancer and as 2022 for those who did not develop the event. For participants with no recorded event year, follow-up time was adjusted to ensure a minimum value of 1 year.

Key systemic health markers were categorised to facilitate analysis. C-reactive protein (CRP) levels were dichotomized into two categories, high (>15 mg/L) and normal (≤15 mg/L), with this binary variable created only for participants with available CRP data. Similarly, estimated glomerular filtration rate (eGFR) was classified based on clinical guidelines as normal (≥60 mL/min/1.73 m^2^) or indicative of kidney disease (<60 mL/min/1.73 m^2^).

After processing, the dataset consisted of 4833 unique participants and 23,742 hospital records, all free from HNC at baseline and with complete follow-up information for survival analysis. This robust dataset, based on physician-diagnosed ICD-10 codes, enabled accurate categorization and reliable analysis of systemic and oral health conditions.

As this study relies on retrospective hospital records, the potential for unmeasured confounding cannot be excluded, particularly regarding lifestyle factors such as smoking and alcohol consumption, which were not systematically recorded in the database. Additionally, while ICD-10 coding provides standardised diagnostic criteria, it lacks granularity in disease severity and progression, potentially leading to residual misclassification bias. To minimise these methodological constraints, we applied strict inclusion criteria, used validated time-to-event models, and accounted for key systemic health markers in the analysis to improve internal validity.

### 4.2. Statistical Analysis

#### 4.2.1. Baseline Characteristics

The baseline characteristics of the study population were summarised to describe the cohort at the start of follow-up. Continuous variables, such as age, were presented as means and standard deviations, in addition to medians and interquartile ranges. Categorical variables, including periodontitis, systemic health markers like CRP and eGFR, and demographic factors like sex, along with other covariates included in the analysis, were summarised as counts and percentages.

#### 4.2.2. Log-Rank Tests and Kaplan–Meier Survival Analysis

The first step in survival analysis involved evaluating differences in survival probabilities across categories of key predictors using the Kaplan–Meier method [67]. Survival curves were generated to visually estimate and compare survival probabilities over time. The figures generated were limited to those covariates that exhibited statistical significance in the final model. The Kaplan–Meier survival function is defined as follows:

(1) $$\hat{s}\left(t\right)=\prod_{t_{i}\leq t}\left(1-\frac{d_{i}}{n_{i}}\right)$$

where $t_{i}$ represents observed event times, $d_{i}$ is the number of events at $t_{i}$, and $n_{i}$ is the number of participants at risk immediately before $t_{i}$. Differences between survival curves were assessed using the log-rank test [68], which evaluates the null hypothesis that survival distributions across groups are identical. The test statistic is calculated as follows:

(2) $$X^{2}=\sum \frac{{\left(O_{i}-E_{i}\right)}^{2}}{E_{i}}$$

where $O_{i}$ represents the observed number of events in group i, and $E_{i}$ is the expected number of events if the survival times were identical across groups. Significant results suggest differences in survival curves between groups. Statistical significance was determined at a threshold of *p* < 0.05, indicating differences in survival probabilities.

#### 4.2.3. Cumulative Hazard Estimation

Cumulative hazard plots were generated using the Nelson–Aalen estimator, a non-parametric method that provides an estimate of the cumulative hazard over time [69,70]. This estimator is defined as follows:

(3) $$\hat{H}\left(t\right)=\sum_{t_{i}\leq t}\frac{d_{i}}{n_{i}}$$

where $\hat{H}\left(t\right)$ is the cumulative hazard at time t, $d_{i}$ represents the number of events (failures) at time $t_{i}$, and $n_{i}$ denotes the number of individuals at risk immediately before $t_{i}$. The cumulative hazard function is a useful tool to visualise the aggregate risk of the event over the study period, providing a complementary perspective to Kaplan–Meier survival curves by focusing on the accumulation of risk rather than the probability of survival. The plots incorporated 95% confidence intervals to account for variability in the hazard estimates.

#### 4.2.4. Weibull Regression

To quantify the effect of systemic and oral health markers on oral cancer risk, parametric survival regression models were employed, with the Weibull distribution chosen for the primary analysis [71,72]. The Weibull model was selected for its flexibility in accommodating hazard rates that change over time, allowing for hazards that either increase or decrease as a function of time. The hazard function for the Weibull model is expressed as follows:

(4) $$h\left(t|X\right)=\lambda \gamma t^{\gamma -1}exp(X^{\prime }\beta )$$

where $h\left(t|X\right)$ represents the hazard rate at time t, λ is the scale parameter, γ is the shape parameter, X is the vector of covariates, and β represents the coefficients. The corresponding survival function is as follows:

(5) $$S\left(t|X\right)=\exp\;(-\;\lambda t^{\gamma }exp(X^{\prime }\beta ))$$

The parameters γ and λ were estimated along with covariate effects β to determine the hazard ratios (HRs) and their 95% confidence intervals (CIs). Significant predictors of oral cancer included systemic health markers, such as high CRP levels and impaired eGFR, as well as periodontitis, all of which were retained in the final model.

The Weibull model estimates the hazard of HNC at any given time based on systemic and oral health markers, allowing for time-dependent risk assessment rather than assuming a constant hazard over follow-up. Unlike the Cox proportional hazards model, which assumes a constant hazard ratio over time (proportional hazards assumption), the Weibull model accommodates varying hazard rates by incorporating a shape parameter (γ). This flexibility enables the identification of whether HNC risk increases (γ>1) or decreases (γ<1) over time, making it particularly suitable for diseases with evolving risk dynamics, such as inflammation-driven malignancies.

#### 4.2.5. Model Comparison

To validate the choice of the Weibull distribution, alternative parametric models were fitted, including the exponential, log-normal, and log-logistic distributions. Additionally, the Weibull model was compared to the semi-parametric Cox proportional hazards model. Model fit was assessed using the Akaike Information Criterion (AIC) [73] and Bayesian Information Criterion (BIC) [74]. Lower AIC and BIC values indicated better model fit. Among the models evaluated, the Weibull regression demonstrated the lowest AIC and BIC values, confirming its suitability for the data.

#### 4.2.6. Model Validation

The predictive performance of the Weibull model was evaluated using receiver operating characteristic (ROC) curves [75] and Harrell’s C-index [76]. The ROC curve quantified the ability of the model to distinguish between participants who developed oral cancer and those who did not. The area under the curve (AUC) provided a summary measure of discrimination, with higher values indicating better predictive ability. Harrell’s C-index, a concordance measure for survival data, assessed the agreement between predicted and observed survival times.

A two-tailed significance threshold of *p* < 0.05 was used for all statistical tests and models throughout the analysis. All statistical analyses and visualisations were performed using Intercooled Stata v18 [77].

## 5. Conclusions

Our findings suggest elevated CRP as a potential adjunctive biomarker for stratifying HNC risk, particularly in individuals with chronic oral inflammatory conditions. The significantly increased hazard observed among patients with periodontitis suggests that this population should be reclassified as high-risk, warranting targeted surveillance and earlier diagnostic interventions. Given CRP’s cost-effectiveness and accessibility in routine laboratory panels, its integration into multifactorial HNC risk prediction models could enhance early detection, particularly in resource-limited settings where comprehensive oncologic screening is unfeasible.

The observed association between structural oral abnormalities, including embedded or impacted teeth, and HNC risk outlines the necessity of comprehensive dental assessments as part of oncologic risk stratification. These findings reinforce the imperative of interdisciplinary collaboration between dental and medical professionals, advocating for the inclusion of systemic inflammatory markers and periodontal health assessments in routine clinical evaluations. Future research should validate CRP’s predictive utility in prospective cohort studies and explore its potential integration with other systemic and molecular biomarkers to refine early detection strategies.

## Figures and Tables

**Table 1:** Baseline characteristics of participants (N = 4833).

Variable	Category	N (%)
Age, years	Mean (SD)	50.43 (15.40)
	Median (IQR)	51 (40–62)
Sex	Male	2193 (45.40)
	Female	2637 (54.60)
Periodontitis	No	3319 (68.67)
	Yes	1514 (31.33)
C-reactive protein	Normal (≤15)	295 (74.31)
	High (>15)	102 (25.69)
eGFR	Normal (≥60)	317 (80.25)
	Kidney Disease (<60)	78 (19.75)
Disorders of tooth development and eruption (K00)	No	4767 (98.63)
	Yes	66 (1.37)
Embedded and impacted teeth (K01)	No	4793 (99.17)
	Yes	40 (0.83)
Dental caries (K02)	No	4698 (97.21)
	Yes	135 (2.79)
Other diseases of hard tissues of teeth (K03)	No	4063 (84.07)
	Yes	770 (15.93)
Diseases of pulp and periapical tissues (K04)	No	4686 (96.96)
	Yes	147 (3.04)
Other disorders of gingiva and edentulous alveolar ridge (K06)	No	4756 (98.41)
	Yes	77 (1.59)
Other specified disorders of teeth and supporting structures (K08)	No	4666 (96.54)
	Yes	167 (3.46)

**Table 2:** Results of the log-rank test for survival distributions by key variables.

Variable	Category	Observed Events	Expected Events	*p*-Value
Periodontitis	No	59	134.85	**<0.001**
	Yes	114	38.15	
C-reactive protein	Normal (≤15)	14	25.9	**0.007**
	High (>15)	20	8.1	
Gender	Male	130	73.5	**<0.001**
	Female	43	99.5	
Disorders of tooth development and eruption (K00)	No	164	168.33	**0.042**
	Yes	9	4.67	
Embedded and impacted teeth (K01)	No	169	171.79	**0.011**
	Yes	4	1.21	
Dental caries (K02)	No	149	164.94	**<0.001**
	Yes	24	8.06	
Other diseases of hard tissues of teeth (K03)	No	129	156.19	**<0.001**
	Yes	44	16.81	
Diseases of pulp and periapical tissues (K04)	No	168	167.22	0.74
	Yes	5	5.78	
Other disorders of gingiva and edentulous alveolar ridge (K06)	No	171	170.95	0.971
	Yes	2	2.05	
Other specified disorders of teeth and supporting structures (K08)	No	148	167.64	**<0.001**
	Yes	25	5.36	
eGFR	Normal (≥60)	13	14.53	0.489
	Kidney Disease (<60)	9	7.47	

The table presents observed and expected events for the log-rank test, along with corresponding *p*-values. Statistically significant differences in survival distributions (*p* < 0.05) are highlighted in bold, suggesting varying survival probabilities across categories.

**Table 3:** Results of Weibull regression analysis for predictors of oral cancer hazard.

Variable	Category	HR (95% CI)	*p*-Value
Periodontitis	Yes vs. No (ref)	**5.99 [1.96–18.30]**	**0.002**
C-reactive protein	High (>15) vs. Normal (≤15, ref)	**4.16 [1.45–12.00]**	**0.008**
eGFR	Kidney Disease (<60) vs. Normal (≥60, ref)	0.95 [0.35–2.61]	0.919
Age	Continuous (per year)	0.98 [0.95–1.02]	0.347
Gender	Female vs. Male (ref)	**0.06 [0.01–0.50]**	**0.009**
Disorders of tooth development and eruption (K00)	Yes vs. No (ref)	3.97 [0.88–17.92]	0.073
Embedded and impacted teeth (K01)	Yes vs. No (ref)	**12.52 [2.48–63.18]**	**0.002**
Dental caries (K02)	Yes vs. No (ref)	2.21 [0.56–8.75]	0.259
Other diseases of hard tissues of teeth (K03)	Yes vs. No (ref)	0.60 [0.13–2.86]	0.524
Diseases of pulp and periapical tissues (K04)	Yes vs. No (ref)	0.52 [0.06–4.61]	0.558
Other specified disorders of teeth and supporting structures (K08)	Yes vs. No (ref)	0.76 [0.18–3.17]	0.704

HR = hazard ratio; CI = confidence interval; ref = reference category. Statistically significant results (*p* < 0.05) are highlighted in bold.

**Table 4:** Shape parameter (*p*) from Weibull regression and its interpretation.

Parameter	Estimate (95% CI)	Interpretation
*p*	2.20 [1.39–3.49]	Hazard increases over time (positive ageing effect)

The parameter estimate indicates the shape of the hazard function over time. A value greater than 1 suggests that the hazard increases with time, indicative of a positive ageing effect.

## Data Availability

The data presented in this study are available on request from the corresponding author. The data are not publicly available due to institutional restrictions.
